# A High-Performance Fluorescence Immunoassay Based on the Relaxation of Quenching, Exemplified by Detection of Cardiac Troponin I

**DOI:** 10.3390/s16050669

**Published:** 2016-05-10

**Authors:** Seung-Wan Kim, Il-Hoon Cho, Ji-Na Park, Sung-Min Seo, Se-Hwan Paek

**Affiliations:** 1 Department of Bio-Microsystem Technology, Korea University, 145 Anam-ro, Sungbuk-gu, Seoul 02841, Korea; kimseungwan@korea.ac.kr (S.-W.K.); gina4664@naver.com (J.-N.P.); ohyeh81@hotmail.com (S.-M.S.); 2 Department of Biomedical Laboratory Science, College of Health Science, Eulji University, Seongnam 13135, Korea; chiuchun0716@gmail.com; 3 Department of Biotechnology and Bioinformatics, Korea University, 2511 Sejong-ro, Sejong 30019, Korea

**Keywords:** signal enhancement, fluorescence quenching, enzymatic fragmentation, photothermal local heating, immunoassay for cardiac troponin I

## Abstract

The intramolecular fluorescence self-quenching phenomenon is a major drawback in developing high-performance fluorometric biosensors which use common fluorophores as signal generators. We propose two strategies involving liberation of the fluorescent molecules by means of enzymatic fragmentation of protein or dehybridization of double-stranded DNA. In the former, bovine serum albumin (BSA) was coupled with the fluorescent BODIPY dye (Red BSA), and then immobilized on a solid surface. When the insolubilized Red BSA was treated with proteinase K (10 units/mL) for 30 min, the fluorescent signal was significantly increased (3.5-fold) compared to the untreated control. In the second case, fluorophore-tagged DNA probes were linked to gold nanoparticles by hybridization with capture DNA strands densely immobilized on the surface. The quenched fluorescence signal was recovered (3.7-fold) by thermal dehybridization, which was induced with light of a specific wavelength (e.g., 530 nm) for less than 1 min. We next applied the Red BSA self-quenching relaxation technique employing enzymatic fragmentation to a high-performance immunoassay of cardiac troponin I (cTnI) in a microtiter plate format. The detection limit was 0.19 ng/mL cTnI, and the fluorescent signal was enhanced approximately 4.1-fold compared with the conventional method of direct measurement of the fluorescent signal from a non-fragmented fluorophore-labeled antibody.

## 1. Introduction

Immunoassays are a unique and well-established methodology for the detection of disease biomarkers. Cardiac troponin I (cTnI) is a typical biomarker used for early diagnosis of acute myocardial infarction, which is crucial for prompt treatment [1]. For immunoassays, enzymes are the most popular tracer for sensitive signal generation using techniques such as colorimetry, chemiluminometry, and electrochemistry [2]. However, proteinaceous tracers are usually labile to thermal energy and tend to deteriorate during long-term storage. This limits their usability as tracers for enzyme-based biosensors.

Fluorescent molecules have advantages in terms of sensitivity and stability over other signal generators, including enzymes [3]. Proteins can be labeled with fluorophores by conjugation, which enables their use in imaging, cell sorting, and biosensing [4]. However, fluorescent tracers are limited by intra-molecular self-quenching upon excitation of neighboring fluorophore molecules separated by a certain distance (e.g., <5 nm; [5]). This quenching effect reduces fluorescent quantum yield and therefore markedly decreases the fluorescent signal [6]. This phenomenon is particularly evident in antibodies labeled with a fluorophore at a high density or use of a high concentration of labeled antibody in an immunoassay. Therefore, this drawback must be overcome to enable use of fluorescent dyes in high-performance analysis.

In this study, for sensitive detection of biomarkers, we resolved the fluorescence-quenching problem by disrupting fluorophore molecules attached to a carrier. To prepare the tracer, bovine serum albumin was adopted as a carrier protein and chemically coupled with a fluorescent dye. Gold nanoparticles were also employed as another type of carrier on which a fluorophore-labeled DNA probe strand was hybridized to the complimentary capture strand immobilized on the surface. For signal production, the quenched state was relaxed by enzymatic digestion of the protein tracer or thermal dehybridization with light of a specific wavelength. Feasibility tests were followed by performance analysis using a microtiter plate-based immunoassay. 

## 2. Materials and Methods

### 2.1. Materials

Human cardiac troponin I (cTnI) and monoclonal antibodies specific to cTnI (clones 19C7 and 560) were purchased from Hytest (Turku, Finland). N-Hydroxysuccinimide (NHS)-functionalized Dylight 650 fluorescent dye (Dylight 650 NHS ester) and DQ^TM^ Red BSA were obtained from Thermo Fisher Scientific (Waltham, MA, USA). Casein (sodium salt), Tween 20, bovine serum albumin (BSA), Sephadex G-15, dialysis tubing, and proteinase K were obtained from Sigma (St. Louis, MO, USA). Succinimidyl-6-[biotin-amido] hexanoate (NHS-LC-Biotin), succinimidyl 4-(N-maleimidomethyl) cyclohexane-1-carboxy-(6-amidocaproate) (LC-SMCC), succinimidyl-6-[3(2-pyridyldithio) propionamido]hexanoate (LC-SPDP) and dithiothreitol (DTT) were purchased from Pierce (Rockford, IL, USA). Black 96-well plates were purchased from Greiner Bio-one (Frickenhausen, Germany). Vivanspin™ 500 was purchased from Sartorius (Goettingen, Germany). Other reagents used in this study were of analytical grade.

### 2.2. Protein Fragmentation-Based Quenching Relaxation

#### 2.2.1. Conjugation of Antibody or SA with Red BSA

Red BSA was first treated with 20-molar excess of LC-SMCC at room temperature (RT) for 2 h. Anti-cTnI monoclonal antibody (clone 19C7) was reacted with 20-fold molar excess of LC-SPDP at RT for 1 h, followed by treatment with 10 mM DTT under the same conditions. The two conjugates activated with maleimide or thiol, respectively, were fractionated by gel filtration using a Sephadex G-15 column (volume, 10 mL) pre-equilibrated with 10 mM phosphate buffer, pH 7.4, containing 150 mM NaCl (PBS). The Red BSA and antibody fractions, the concentrations of which were determined by Bradford assay [7], were immediately mixed at a molar ratio of 5 and reacted at RT overnight (>12 h). An identical procedure was applied for conjugation of Red BSA with SA.

#### 2.2.2. Protease-Mediated Red BSA Fragmentation

To evaluate protease-assisted protein fragmentation, Red BSA was first immobilized on the surface of wells of a black microtiter plate via SA-biotin linkage. BSA (1 g/mL) reacted with 20-molar excess of NHS-LC-Biotin as described elsewhere [8] was bound to the microwell surfaces and unbound surfaces were blocked with 5% BSA in PBS (BSA-PBS). Red BSA conjugated with SA diluted in BSA-PBS to 0–4.35 g/mL was added to the treated microwells, followed by incubation at 37 °C for 1 h. The fluorescence signal intensity was measured at an excitation wavelength of 590 nm and emission wavelength of 620 nm using a microplate reader (Synergy™ H4, BioTek Inc.; Winooski, VT, USA). The same procedure was repeated with addition of proteinase K (10 units/mL) treatment at RT for 30 min. 

#### 2.2.3. Optimization of Immunoassay Performance

To utilize the Red BSA-conjugated antibody (19C7-Red BSA) as a detection binder, a sandwich immunoassay for cTnI was carried out using a microtiter plate as the solid matrix. The capture antibody (clone 560; 100 µL of 1 g/mL) in PBS was coated on the surfaces of microwells by incubation in a humidity chamber at 4 °C overnight (12 h). After washing three times with deionized water, the residual surfaces were blocked with PBS containing 0.5% casein (casein-PBS, 200 L), and then incubated at 37 °C for 2h. After a second washing, various concentrations of cTnI and the Red BSA-conjugated detection antibody (100 L of 2 g/mL) diluted in casein-PBS containing 0.1% Triton X-100 (casein-PBS-Triton) were sequentially reacted to form a sandwich complex at RT for 2 h. After the final washing, fluorescence signal intensity from the sandwich complex was measured at a specific wavelength using a microtiter plate reader as described above. For optimization, the same procedure was repeated, with the exception of treatment with 0.1–10 units/mL proteinase K for 30 min. The mean of triplicate measurements was used to plot the dose-response curve; standard deviations were also indicated. The detection limit was determined as the analyte concentration corresponding to a signal value that was three-fold larger than the standard deviation of the signal at the zero dose. Using 10 units/mL proteinase K, the treatment condition was further tested by varying the digestion time in the range 0–120 min.

### 2.3. DNA Dehybridization-Based Quenching Relaxation 

#### 2.3.1. Preparation of ssDNA-Grafted Gold Nanoparticles

Gold nanoparticles (AuNP), 41.1 nm in diameter, were synthesized via citrate reduction of chloroauric acid [9], and their size was determined using a NanoBrook 90 Plus Particle Size Analyzer™ (Brookhaven Instruments, Holtsville, NY, USA). For immobilization of capture ssDNA strands (HS-CC-5′-TCTTTTGTCTTATGTTCTTCG-3′), AuNP (1 mL) was first reacted with 5% casein dissolved in 100 mM phosphate buffer, pH 7.4, (100 L) under gentle shaking at RT for 12 h. After centrifugation at 13,000 rpm for 15 min, the supernatant was discarded and the pellet resuspended in PBS; this procedure was repeated three times. LC-SMCC(25 g) was reacted with casein-coated nanoparticles at RT for 1 h to induce maleimide activation, followed by centrifugation at 11,000 rpm for 10 min. Immediately after resuspension of the pellet in PBS (500 L), the capture strand (1 nmol) terminated by a thiol functional group was combined with the maleimide moiety on the AuNP, and incubated at RT for 12 h. Unbound strands were removed by centrifugation and the conjugate was characterized using an ND-1000 Nanodrop Spectrophotometer™ (Thermo Fisher Scientific, Waltham, MA, USA).

#### 2.3.2. Hybridization of Dye-Labeled Probe Strands

Probe strand comprising oligonucleotides (21 mers) coupled with Alexa647 at the 3′ end of (5′-CGA AGA ACA TAA GAC AAA AGA-3′-Alexa647) was hybridized with the capture strands pre-immobilized on nanoparticle surfaces. The dye-tagged probe strand (1 nmol) was mixed with the capture strand on the gold nanoparticles (500 L) at 60 °C for 1 h and residual probe strand was removed by centrifugation at 11,000 rpm for 10 min and resuspension in 1 M saline sodium citrate buffer (SSC); this procedure was repeated three times.

#### 2.3.3. Temperature-Controlled Dehybridization

To evaluate quenching relaxation, the dye-labeled probe strand was released from the hybridization by thermal denaturation of the dsDNA structure at the appropriate melting temperature. AuNPs coupled with the dye-linked probe strand were diluted 100-fold in 1 M SSC, and an aliquot (100 L) was added to a black 96-well microtiter plate. The plate was heated to 60 °C or 80 °C in a heating chamber and the fluorescence signal was measured at an excitation wavelength of 650 nm and emission wavelength of 675 nm using a microplate reader. Alternatively, the complex was irradiated with light of 530 nm wavelength for 1min to induce a photothermal effect, and subjected to the measurement procedure described above.

### 2.4. Conventional Fluorescent Immunoassay

#### 2.4.1. Conjugation of Antibody to Fluorescent Dye

A monoclonal antibody (clone 19C7) was labeled with fluorescent dye (Dylight650 NHS ester) following the manufacturer’s instructions. The succinimidyl ester moiety of the dye reacts with primary amines on the antibody, forming a stable dye-labeled conjugate. Briefly, antibody (300 g) in PBS was reacted with a 7-molar excess of the dye dissolved in dimethyl sulfoxide and incubated at RT for 2 h. Free dye molecules in the mixture were separated by gel filtration through a Sephadex G-15 column (volume 10 mL) which was pre-equilibrated in PBS. The synthesized conjugate was dialyzed twice in PBS and then concentrated via ultra-centrifugation using a Vivaspin™ 500. The dye-coupled antibody (1.7 mg/mL concentration) was stored at 4 °C.

#### 2.4.2. Analytical Procedure

A conventional immunoassay was carried out using a black microtiter plate according to a standard protocol [10]. The capture antibody (clone 560; 100 L of 1 g/mL) was immobilized on microwell surfaces as described for the immunoassay using the Red BSA-conjugated detection antibody. After washing, various concentrations of cTnI and the Dylight650-labeled detection antibody (clone 19C7; 100 L of 2 g/mL) diluted in Casein-PBS-Triton were sequentially reacted at RT for 2 h. After the final washing, PBS (100 L) was added and the fluorescence signal was measured at an excitation wavelength of 650 nm and emission wavelength of 665 nm using a microplate reader. The remaining analytical steps were identical to those described earlier.

## 3. Results and Discussion

### 3.1. Analytical Concepts

#### Definitions of Terms

**Figure 1:**
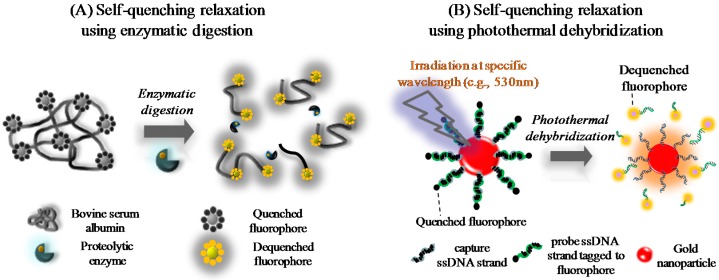
Signal generation from carrier-bound fluorescent dye molecules by reduction of the quenching phenomenon. Use of dye molecules heavily coupled with a carrier protein (e.g., BSA) resulted in relaxation of self-quenching at the time of signal generation by protease digestion (**A**). Nanoparticles can also be used as carriers by linking via DNA hybridization. Upon excitation, the fluorophore excitation energy is transferred to the surface of the plasmonic metal. This quenching may be relaxed by irradiation, which produces photothermal energy that dehybridizes the linker DNA prior to signal generation (**B**).

The proposed immuno-analytical strategies based on protease-assisted or light-induced signal generation from fluorophore molecules densely coupled with a carrier are depicted in Figure 1. For protease-assisted signal generation, BSA was conjugated to several dye molecules (e.g., BODIPY) through its functional amino acid residues, such as lysine and cysteine [11]. BSA was chemically activated and then heavily coupled with fluorescent BODIPY dye (Red BSA). In such a situation, adjacent dye molecules can influence each other by energy transfer upon excitation with incident light [12]. This effect, known as intra-molecular self-quenching, reduces the overall fluorescent signal. Digestion with the broad-spectrum serine protease proteinase K [13], results in generation of small peptide fragments. The fluorescence signal increases with increasing digestion time due to recovery of the fluorescence of individual dye molecules coupled to the digested fragments.

Nanoparticles can be used as carriers of fluorophore molecules due to their large surface area. However, the fluorescence energy excited by an exterior light source can be transferred to plasmonic gold or silver nanoparticles [14]. The fluorescence signal, therefore, tends to be quenched within a certain distance from the nanoparticle surface. This can be resolved by releasing the dye to the bulk solution using the same concept as for the protease-assisted technique. To this end, fluorophores and nanoparticles were chemically conjugated with DNA single-stranded oligomers (ssDNA) and then tethered by hybridization. When nanoparticles are irradiated with light close to the maximum absorption wavelength, they emit heat from the surfaces and increase local temperature, which is known as the photothermal effect. The fluorophore-bearing probe strands are dehybridized when the melting point of the double-stranded DNA (dsDNA) is reached and the fluorophores are liberated, which enables recovery of the intrinsic fluorescence signal upon excitation.

### 3.2. Preliminary Experimental Models

#### 3.2.1. Protease-Assisted Protein Fragmentation Model

**Figure 2:**
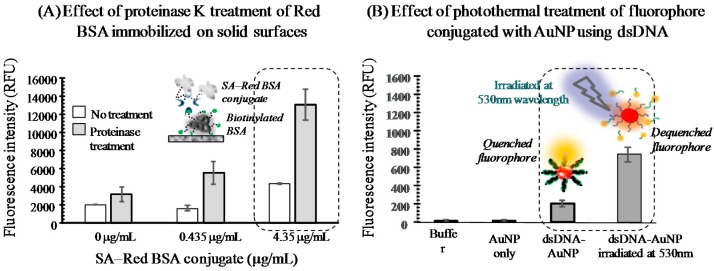
Preliminary tests of the two concepts shown in Figure 1. To evaluate protease-assisted protein fragmentation, a solid substrate (e.g., microwell) was coated with Red BSA via biotin-SA linkage (**A**). At Red BSA concentrations ≥4.35 g/mL, the fluorescence signal increased compared to the background (0 g/mL; white bars). The same preparations were then treated with proteinase K for 30 min, and exhibited elevated signals relative to those without treatment (white *vs.* grey bars). To examine photothermal energy-induced dehybridization, gold nanoparticles (e.g., 41.1 nm diameter; AuNP) were chemically coupled with ssDNA and then hybridized with a fluorophore-labeled, complementary single DNA (**B**). The fluorescence signal from the modified AuNP (third bar) was higher than the background (first and second bars). Irradiation of AuNPs in solution at the maximum absorbance wavelength (e.g., 530 nm) resulted in a marked increase in signal upon excitation (fourth bar).

To prove the concept proposed in Figure 1A, Red BSA was immobilized on a solid substrate and then treated with proteinase K to monitor the fluorescence signal change (Figure 2A). The inner surfaces of microwells were coated with biotinylated BSA and Red BSA conjugated with SA (SA-Red BSA) was then immobilized through a biotin-SA linkage. As the amount of SA-Red BSA conjugate increased, the fluorescence signal remained at the background level at a low dose of conjugate (e.g., 0.435 g/mL). 

In contrast, the fluorescence signal was significantly augmented at a 10-fold increased dose (Figure 2A; white bars). To determine the fragmentation effect, the experiments were repeated with protease digestion for 30 min (Figure 2A; grey bars). Although no SA-Red BSA was immobilized (0 g/mL), the signal was elevated about 1.5-fold compared to that without protease treatment. As the SA-Red BSA conjugate immobilization increased, the ratio increased 3–3.5-fold at >0.435 g/mL conjugate. Therefore, protease-assisted BSA fragmentation enhanced the fluorescence signal from dye molecules coupled with a carrier protein.

#### 3.2.2. Photothermal Energy-Induced Dehybridization Model

**Figure A1:**
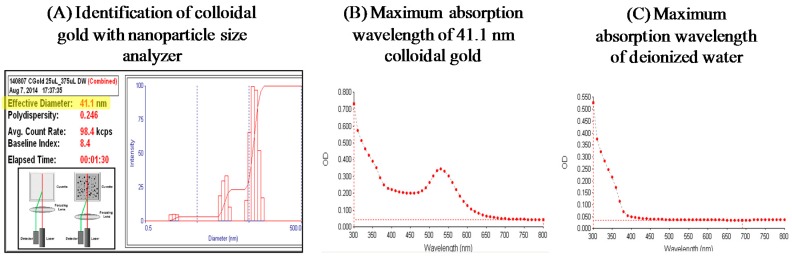
Synthesis of gold nanoparticles of uniform size and characterization of their size and maximum wavelength. The particles were generated using the citrate reduction method and had an effective diameter of ~41.1 nm (A). The maximum absorption wavelength of the particles was ~530 nm by comparison of the absorbance spectrum (B) with that of deionized water (C).

As mentioned above, the fluorescence signal quenched near nanoparticle surfaces can be enhanced by the local temperature increase induced by photothermal energy produced upon irradiation with light of the wavelength maximally absorbed by the nanoparticle [15]. To prepare a light-responsive carrier, gold nanoparticles were synthesized via citrate reduction and characterized by dynamic light scattering [16], which revealed that the nanoparticles were of 41.1 nm effective diameter (Figure A1A). The maximum absorption peak was at 530 nm (Figure A1B), compared to that for deionized water as background (C). The gold particles were coated with a protein to adopt fluorophore-labeled dsDNA (FL-dsDNA), which was carried out by chemically linking each strand to a fluorophore and nanoparticle, followed by hybridization. The coupling of dsDNA to the gold particles was verified via zeta potential measurement (data not shown; [17]). The negative charge of bare gold was slightly shifted in a positive direction by the final modification step (e.g., −47 to −38 mV), possibly due to masking of the surface via adsorption of modifiers such as protein and DNA probes. Wavelength was red-shifted and no aggregation occurred upon addition of sodium chloride. These results are consistent with previous reports [18].

**Figure A2:**
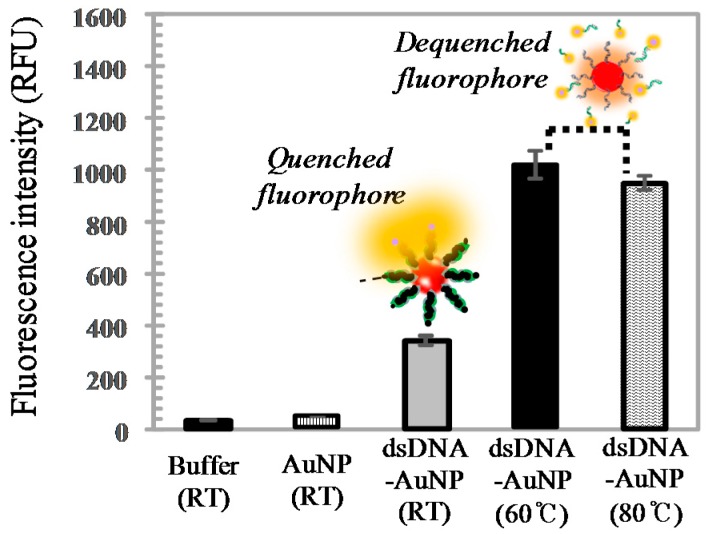
Effect of temperature on signal generation from fluorescent dyes coupled to gold nanoparticles via dsDNA (FL-dsDNA-AuNP). The fluorescence signal of the modified nanoparticles in solution was low at RT upon excitation (third bar), but was higher than the background from buffer or gold (first and second bars). When FL-labeled AuNPs were exposed to water at higher temperatures (fourth and fifth bars), the signals were significantly increased compared to that at RT. This was because the heat energy dehybridized the dsDNA strands, resulting in release of the probe into the bulk solution.

Thermal dehybridization of the FL-dsDNA-grafted nanoparticles (FL-dsDNA-AuNP) was examined by treatment with high-temperature water. The fluorescence signal was initially low at RT upon excitation, which was caused by quenching due to energy transfer to the nanoparticle (Figure A2 third bar). However, the level was higher than the background (Figure A2, first and second bars). This implied that fluorophores at the ends of probe strands were not completely quenched. When FL-dsDNA-AuNPs were exposed to 60 and 80 °C, the signal increased significantly compared to that at RT (Figure A2, fourth and fifth bars, respectively). Under these conditions, the dsDNA could be dehybridized and the probe strand with the fluorophore released into the bulk solution. This enabled the fluorophore to recover its intrinsic property (*i.e.*, dequenching). Since no increase in signal was detected in water at 80 °C compared to that at 60 °C, the lower temperature was optimal.

The photothermal effect was evaluated by irradiating FL-dsDNA-AuNP in solution at a wavelength of 530 nm (the maximum absorbance of the gold nanoparticles) and monitoring the fluorescence signal in a microtiter plate reader (Figure A1B). A fluorescence signal from the FL-dsDNA-AuNP was detected upon excitation in the absence of treatment (Figure 2B; third bar), which was higher than the background (first and second bars), due to partial quenching of the fluorophore. The signal increased 3.7-fold upon application of irradiation compared to that without treatment (Figure 2B; fourth bar). This was due to dehybridization of dsDNA caused by the increased local temperature. 

We next performed high-performance fluorescence immunoassay based on relaxation of the quenched signal via protease-assisted protein fragmentation since this technique was relatively simple in antibody labeling. The target analyte in this study was cTnI, a specific biomarker of acute myocardial infarction, which requires a high-sensitivity assay for early detection [19].

### 3.3. Immunoassay Employing Protease-Assisted Signal Enhancement

To construct a fluorometric immuno-analytical system for cTnI, an anti-cTnI detection antibody (clone 19C7) was coupled directly to Red BSA by chemical cross-linking and the capture antibody (clone 560) was immobilized on the surface of wells of a microtiter plate. Enzymatic digestion was performed using the optimal enzyme concentration and reaction time at RT. cTnI immunoassay was carried out by forming a sandwich immuno-complex in the following reaction order: the capture antibody, analyte, and detection antibody coupled to Red BSA.

#### 3.3.1. Optimization for Fluorescent Signal Generation

**Figure 3:**
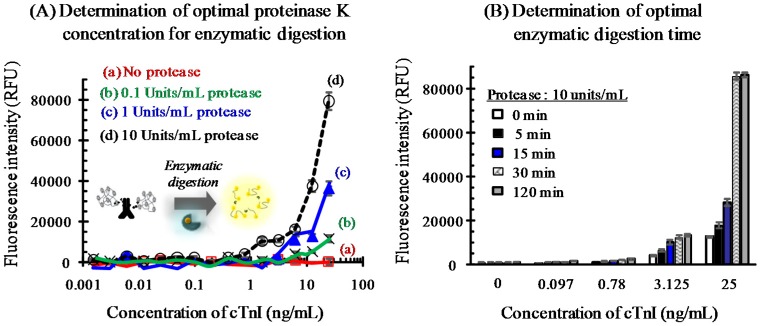
Determination of optimal conditions for the enzymatic digestion of Red BSA. The proteinase K concentration was varied from 0–10 units/mL with a 30 min reaction (**A**). The dose-response curves obtained showed that although little fluorescent signal was observed in the absence of protease (no protease), the signal at a constant cTnI dose increased with increasing protease concentration from 0.1 to 10 units/mL. Using the optimum protease concentration of 10 units/mL the detection limit was ~0.19 ng/mL cTnI. The digestion time was then optimized in the range 0–120 min (**B**). The fluorescent signal measured at a constant analyte dose increased over time, and was significantly enhanced at 25 ng/mL cTnI when the reaction time was ≥30 min. Therefore, digestion was carried out using 10 units/mL proteinase K for 30 min.

To determine the optimal concentration of proteinase K, immunoassays were performed using 0–10 units/mL protease and a 30 min reaction, and dose-responses curves were obtained by plotting the signal against the cTnI concentration (Figure 3A). The fluorescence intensity differed significantly according to the protease concentration. In the absence of protease, no signal was observed irrespective of analyte concentration (Figure 3A, No protease), suggesting complete quenching of the fluorophore. Under this condition, the fluorophore molecules were positioned within the energy transfer distance (*i.e.*, Forster resonance energy transfer (FRET) distance). In contrast, the fluorescence signal increased with increasing enzyme concentration from 0.1 to 10 units/mL. Although this effect was greater at higher doses of cTnI, relaxation of the quenched signal may also be used to detect analyte at lower concentration. Indeed, the detection limit was reduced to 0.19 ng/mL cTnI when 10 units/mL protease was used. 

Next, the digestion time was optimized by in a range of 0 to 120 min (Figure 3B). Although the signal increased up to 30 min, it increased dramatically at >30 min (compare the response signals to 25 ng/mL cTnI). Therefore, the fluorophore molecules in Red BSA were liberated by enzymatic digestion and released into the bulk solution. This rendered the influence of proximity, *i.e.*, self-quenching, among the fluorescent molecules negligible. 

#### 3.3.2. Immunoassay Performance

Using the optimal digestion conditions (*i.e.*, 10 units of proteinase K and a 30 min reaction), the fluorescent signal was produced through relaxation of fluorophore self-quenching. The detection capability of the protease-assisted method for cTnI using the Red-BSA as label was then compared with that of a conventional immunoassay. In the latter, a fluorophore-labeled detection antibody was used to generate signal upon excitation. A detection antibody (19C7) specific to the analyte was chemically coupled to an activated fluorophore (e.g., Dylight650) at a limited ratio (usually, 3–9 dye molecules per immunoglobulin unit) to minimize adverse effects on antigen-antibody binding [20].

**Figure 4:**
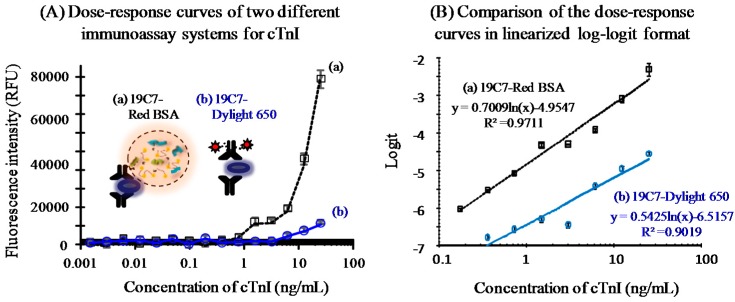
Analytical performance of two fluorometric immunoassay systems employing different signal generation techniques. The novel immunoassay based on protease treatment of Red-BSA was performed using the optimal digestion conditions (Figure 3). The dose-response curve showed a detection limit of 0.19 ng/mL cTnI (**A**, a). A conventional immunoassay, in which the same detection antibody was labeled with Dylight650 fluorophore, was also employed (**A**, b). The dose-response curve showed that the detection capability (0.78 ng/mL) was ~4.1-fold lower than that of the protease-assisted system. The two curves were linearized using log-logit transformation, which demonstrated the novel technique to have higher performance than the conventional approach (**B**).

The dose-response curves of the two systems showed that the protease-treated system produced markedly higher signals over the overall cTnI dose range than the conventional assay (Figure 4A). Such signal amplification yielded 4.1-fold enhancement of the detection limit with the former (0.19 ng/mL) compared to the latter (0.78 ng/mL). The detection limit was calculated by multiplying the standard deviation at the zero dose by three and obtaining the cTnI dose corresponding to the signal value. The curves were then linearized via log-logit transformation and regressed to obtain linear equations with R^2^ > 0.97 (Figure 4B). This indicated that the novel system had higher sensitivity than the conventional assay. Such inferior performance of the traditional system may be due mainly to the limited number of fluorescent dyes coupled to the antibody molecule. Although the amount of labeled antibody can be increased to improve the sensitivity, this approach also leads to quenching due to the proximity of the antibody molecules on the solid surface. In contrast, the protease-assisted method alleviated the distance problem by means of forced diffusion of the dye by fragmentation into the bulk solution, particularly outside of the FRET distance [21]. In case of real sample test using serum or plasma, the analysis is usually carried out by diluting the sample to a range of 1/2−1/10 with a defined medium to control non-specific binding. Other analytical conditions are remained identical to those described earlier. Therefore, under such analytical conditions, the relative performance of the proposed and conventional systems will not be significantly different from those shown in this study.

The signal enhancement by relaxing the quenched fluorescence demonstrated in this study was simple to apply to immunoassay system. However, it may impose a burden that the enzymatic digestion required an extra time until a significant signal was produced (e.g., 30 min). Similar concepts of dequenching fluorescence have been reported by other groups employing more sophisticated signal generation techniques. One of them prepared quench-based antibody probes, ‘Quenchbodies’, which can produce fluorescent signal upon antigen binding [22]. Although this approach offered an immediate signal generation after analysis, an intensive effort may be needed to prepare the immuno-reagent through antibody engineering. Another group devised a signal quenching system by coupling quantum dot and graphene, each of which was tethered to antibody and antigen [23]. The signal from quantum dot was initially quenched by antigen-antibody binding and, upon antigen presence in sample, can be produced based on competitive binding. This may be used for multi-analyte detection when different quantum dots are used, but will be limited to competition-based assays usually showing low sensitivity.

## 4. Conclusions

We have proposed two strategies for improving immuno-analytical sensitivity through relaxation of the quenched fluorescence signal from heavily fluorophore-labeled carriers; *i.e.*, BSA or DNA-grafted gold nanoparticles. By simply treating with protease or dehybridizing by irradiation, the dye molecules initially tethered to the carrier were liberated from the energy transfer range and a fluorescent signal was produced upon excitation. These techniques could be used in microtiter plate format immunoassays for detection of biomarkers, such as cTnI, with high sensitivity. Furthermore, protease treatment enhanced the detection capability of cTnI 4.1-fold compared to a conventional assay. This concept may also be applied to other biosensor platforms—such as lateral-flow immunoassays, protein chips, and microfluidic devices—in which the fluorescence signal must be enhanced. A further study is underway to enhance analytical sensitivity by employing gold nanoparticles to enable transport of fluorophore molecules in a self-quenched state.

## Abbreviations

The following abbreviations are used in this manuscript:

Ds-DNA	Double strand-deoxyribonucleic acid
AuNPs	Gold nanoparticles
cTnI	Cardiac troponin I
FRET	Fluorescence resonance energy transfer
BSA	Bovine serum albumin
PBS	Phosphate buffered saline
DTT	Dithiothreitol
RT	Room temperature

## References

[1] Morrow D.A., Cannon C.P., Rifai N., Frey M.J., Vicari R., Lakkis N., Robertson D.H., Hille D.A., DeLucca P.T., DiBattiste P.M. (2001). Ability of minor elevations of troponins I and T to predict benefit from an early invasive strategy in patients with unstable angina and non-ST elevation myocardial infarction: Results from a randomized trial. J. Am. Med. Assoc..

[2] Roberts I.M., Jones S.L., Premier R.R., Cox J.C. (1991). A comparison of the sensitivity and specificity of enzyme immunoassays and time-resolved fluoroimmunoassay. J. Immunol. Methods.

[3] Luppa P.B., Sokoll L.J., Chan D.W. (2001). Immunosensors—principles and applications to clinical chemistry. Clin. Chim. Acta.

[4] Sukhanova A., Devy J., Venteo L., Kaplan H., Artemyev M., Oleinikov V., Klinov D., Pluot M., Cohen J.H., Nabiev I. (2004). Biocompatible fluorescent nanocrystals for immunolabeling of membrane proteins and cells. Anal. Biochem..

[5] Schneider G., Decher G., Nerambourg N., Praho R., Werts M.H., Blanchard-Desce M. (2006). Distance-dependent fluorescence quenching on gold nanoparticles ensheathed with layer-by-layer assembled polyelectrolytes. Nano Lett..

[6] Ao L., Gao F., Pan B., He R., Cui D. (2006). Fluoroimmunoassay for antigen based on fluorescence quenching signal of gold nanoparticles. Anal. Chem..

[7] Bradford M.M. (1976). A rapid and sensitive method for the quantitation of microgram quantities of protein utilizing the principle of protein-dye binding. Anal. Biochem..

[8] Holmes K.L., Lantz L.M. (2001). Protein labeling with fluorescent probes. Methods cell biol..

[9] Bhargava S.K., Booth J.M., Agrawal S., Coloe P., Kar G. (2005). Gold nanoparticle formation during bromoaurate reduction by amino acids. Langmuir.

[10] Bodor G.S., Porter S., Landt Y., Ladenson J.H. (1992). Development of monoclonal antibodies for an assay of cardiac troponin-I and preliminary results in suspected cases of myocardial infarction. Clin. Chem..

[11] Casari G., Sander C., Valencia A. (1995). A method to predict functional residues in proteins. Nature struct. Biol..

[12] Chen R.F., Knutson J.R. (1988). Mechanism of fluorescence concentration quenching of carboxyfluorescein in liposomes: Energy transfer to nonfluorescent dimers. Anal. Biochem..

[13] Ebeling W., Hennrich N., Klockow M., Metz H., Orth H.D., Lang H. (1974). Proteinase K from Tritirachium album Limber. Eur. J. Biochem..

[14] Chen Y., Munechika K., Ginger D.S. (2007). Dependence of fluorescence intensity on the spectral overlap between fluorophores and plasmon resonant single silver nanoparticles. Nano lett..

[15] Mitsudome T., Noujima A., Mikami Y., Mizugaki T., Jitsukawa K., Kaneda K. (2010). Supported gold and silver nanoparticles for catalytic deoxygenation of epoxides into alkenes. Angew. Chem..

[16] Liu X., Dai Q., Austin L., Coutts J., Knowles G., Zou J., Chen H., Huo Q. (2008). A one-step homogeneous immunoassay for cancer biomarker detection using gold nanoparticle probes coupled with dynamic light scattering. J. Am. Chem. Soc..

[17] Arjmandi N., Van Roy W., Lagae L., Borghs G. (2012). Measuring the electric charge and zeta potential of nanometer-sized objects using pyramidal-shaped nanopores. Anal. Chem..

[18] Kim J.H., Cho J.H., Cha G.S., Lee C.W., Kim H.B., Paek S.H. (2000). Conductimetric membrane strip immunosensor with polyaniline-bound gold colloids as signal generator. Biosens. Bioelectron..

[19] Reichlin T., Hochholzer W., Bassetti S., Steuer S., Stelzig C., Hartwiger S., Biedert S., Schaub N., Buerge C., Potocki M. (2009). Early diagnosis of myocardial infarction with sensitive cardiac troponin assays. N. Engl. J. Med..

[20] Cho J.H., Kim M.H., Mok R.S., Jeon J.W., Lim G.S., Chai C.Y., Paek S.H. (2014). Two-dimensional paper chromatography-based fluorescent immunosensor for detecting acute myocardial infarction markers. J. Chromatogr. B..

[21] Kohl T., Heinze K.G., Kuhlemann R., Koltermann A., Schwille P. (2002). A protease assay for two-photon crosscorrelation and FRET analysis based solely on fluorescent proteins. Proc. Natl. Acad. Sci. USA.

[22] Abe R., Ohashi H., Iijima I., Ihara M., Takagi H., Hohsaka T., Ueda H. (2011). “Quenchbodies”: Quench-based antibody probes that show antigen-dependent fluorescence. J. Am. Chem. Soc..

[23] Anfossi L., Calza P., Sordello F., Giovannoli C., Di Nardo F., Passini C., Cerruti M., Goryacheva I.Y., Speranskaya E.S., Baggiani C. (2014). Multi-analyte homogenous immunoassay based on quenching of quantum dots by functionalized graphene. Anal. Bioanal. Chem..

